# Systemic Pharmacological Smoothened Inhibition Reduces Lung T-Cell Infiltration and Ameliorates Th2 Inflammation in a Mouse Model of Allergic Airway Disease

**DOI:** 10.3389/fimmu.2021.737245

**Published:** 2021-09-10

**Authors:** Diana C. Yánez, Eleftheria Papaioannou, Mira M. Chawda, Jasmine Rowell, Susan Ross, Ching-In Lau, Tessa Crompton

**Affiliations:** ^1^UCL Great Ormond Street Institute of Child Health, London, United Kingdom; ^2^School of Medicine, Universidad San Francisco de Quito, Quito, Ecuador

**Keywords:** smoothened (SMO), airway inflammation, Th2, Sonic Hedgehog (Shh), CD4 T-cell

## Abstract

Allergic asthma is a common inflammatory airway disease in which Th2 immune response and inflammation are thought to be triggered by inhalation of environmental allergens. Many studies using mouse models and human tissues and genome-wide association have indicated that Sonic Hedgehog (Shh) and the Hedgehog (Hh) signaling pathway are involved in allergic asthma and that Shh is upregulated in the lung on disease induction. We used a papain-induced mouse model of allergic airway inflammation to investigate the impact of systemic pharmacological inhibition of the Hh signal transduction molecule smoothened on allergic airway disease induction and severity. Smoothened-inhibitor treatment reduced the induction of Shh, IL-4, and IL-13 in the lung and decreased serum IgE, as well as the expression of *Smo*, *Il4*, *Il13*, and the mucin gene *Muc5ac* in lung tissue. Smoothened inhibitor treatment reduced cellular infiltration of eosinophils, mast cells, basophils, and CD4+ T-cells to the lung, and eosinophils and CD4+ T-cells in the bronchoalveolar lavage. In the mediastinal lymph nodes, smoothened inhibitor treatment reduced the number of CD4+ T-cells, and the cell surface expression of Th2 markers ST2 and IL-4rα and expression of Th2 cytokines. Thus, overall pharmacological smoothened inhibition attenuated T-cell infiltration to the lung and Th2 function and reduced disease severity and inflammation in the airway.

## Introduction

Allergic asthma is a common inflammatory disease of the lungs and airways in which Th2 immune responses and inflammation are triggered by inhalation of environmental allergens. Sensitization to the allergen leads to Th2 differentiation of naïve CD4+ T-cells, which then further drive disease by secretion of cytokines, leading to IgE, mast cell, basophil, and eosinophil responses, increased mucous production, and in some instances chronic inflammation and airway remodeling (1). Factors that lead to CD4+ Th2 differentiation on activation of naïve CD4+ T-cells and their recruitment to the lung are therefore likely to promote sensitization and disease and may provide possible targets for new therapies.

Many studies using mouse models, human tissues and genome-wide association (GWAS) have indicated that the Sonic Hedgehog (Shh) and Hedgehog (Hh) signaling pathways are involved in allergic asthma (2–8), and Hh signaling to naïve CD4+ T-cells promotes Th2 differentiation *in vitro* in mouse and human (4, 9, 10). Therefore, pharmacological targeting of the Hh pathway might provide a possible treatment strategy for allergic asthma, targeting both airway inflammation and the Th2 differentiation which is believed to drive the disease. However, Hh pathway activation in T-cells has also been shown to impact many different aspects of T-cell function (11–16), in addition to promoting Th2 differentiation, and so it is important to test the impact of pharmacological Hh inhibition in animal models of allergic airway disease.

The three mammalian Hh proteins, Shh, Indian Hh (Ihh), and Desert Hh (Dhh), are intercellular signaling proteins which share a common signaling pathway (17). Hh proteins bind to their cell surface receptor (Patched1) Ptch1, which releases Ptch1’s repression of the signal transduction molecule smoothened (Smo) and Smo signals to activate the downstream transcription factors, Gli1, Gli2, and Gli3. Smo is believed to be a non-redundant component of the pathway and so has been targeted for pharmacological inhibition (18, 19). However, non-canonical Smo-independent Hh pathway activation has been described in some tissues and cells (17).

Components of the Hh pathway, including Ptch1, Smo, Gli1, Gli2, and Ihh, are expressed in T-cells and regulate T-cell development and function (4, 9, 13, 20–22). During T-cell development in the thymus, Shh and Ihh promote the earliest stages of T-cell development but negatively regulate pre-TCR- and TCR-induced differentiation, by reducing TCR signal strength (12, 21, 23–25). In naïve T-cells, Hh pathway activation again reduces TCR signaling to inhibit proliferation and activation on TCR/CD28 ligation (9, 11, 12), but also influences many aspects of T-cell differentiation. In addition to promoting CD4+ Th2 effector differentiation *in vitro* and in allergic airways disease (4, 10), Hh signaling has been shown to drive CD8+ cytotoxic T cell (CTL) differentiation *in vitro* (13) and to promote NK*γ*δ T-cell differentiation and CD4+ regulatory T-cell (Treg) differentiation and function *in vivo* and *in vitro* (14–16, 26–28). Consistent with its role in Tregs, in many tissues Hh signaling has been shown to have anti-inflammatory action (15, 29–35), in contrast to its well-known pro-inflammatory role in allergic asthma (2–8).

In this study, we investigated the impact of systemic pharmacological Smo inhibition on disease induction and severity in a mouse model of allergic airway disease. We showed that Smo inhibition attenuated T-cell infiltration to the lung and Th2 differentiation and reduced disease severity and inflammation in the airway.

## Materials and Methods

Reagents and antibodies used in this study are summarized in **Supplementary Table 1**.

### Mice

C57BL/6 (6–8-week-old) mice were bred at University College London (UCL) from breeding pairs purchased from Envigo and the Jackson Laboratory. Mice were maintained in a specific pathogen-free environment with water and food ad libitum and a regular light–dark cycle. Animal studies were carried out under UK government regulations, following ethical approval at UCL. For ethical considerations, and to avoid the unnecessary breeding of mice that could not be used experimentally, both male and female mice were used in these experiments. Male and female mice were assigned to experimental groups in equal proportion, to prevent gender differences influencing experimental outcome.

### Immunization Protocol

**Figure 1:**
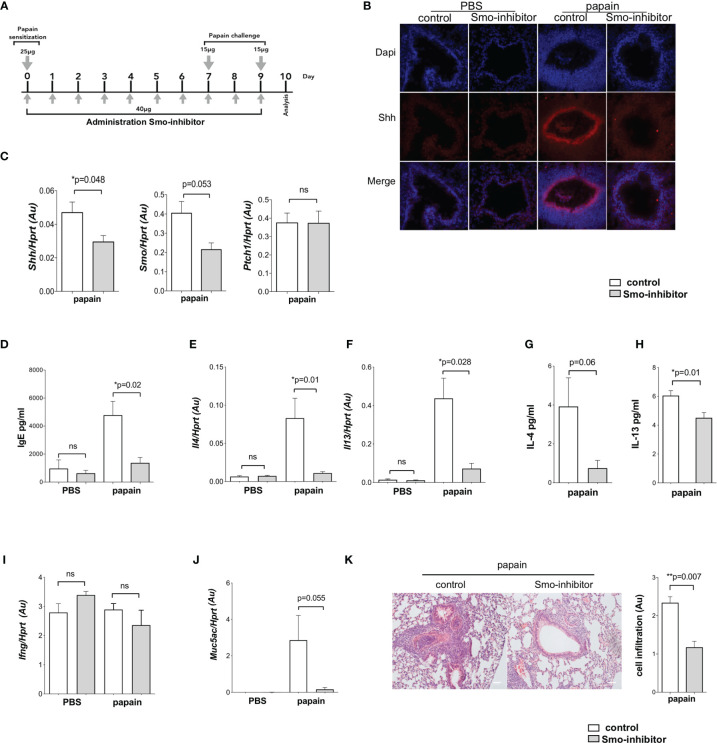
Systemic smoothened-inhibitor treatment reduced the expression of Hh pathway components and the inflammatory response upon allergic sensitization. **(A)** Cartoon illustrates the papain protocol: mice underwent intranasal administration of papain or PBS alone on days 0, 7, and 9, together with a daily intraperitoneal injection of Smo inhibitor or DMSO (control) according to the timeline and doses shown. **(B)** Representative immunofluorescence staining images of Shh expression (red) and DAPI-stained nuclei (blue) in frozen lung sections from the four groups of mice (n = 3 per group). **(C)** Expression (QRT-PCR) of *Shh* (n = 5), *Smo* (n = 3), and *Ptch1* (n = 5) in lung homogenates from control and Smo-inhibitor-treated groups after the papain protocol. **(D)** Blood serum IgE (ELISA) from the four groups of mice. **(E)** *Il4* and **(F)** *Il13* gene expression (mean ± SEM) in lung homogenates of control and Smo-inhibitor groups from PBS and papain protocol by QRT-PCR. **(G, H)** Protein level of **(G)** IL-4 and **(H)** IL-13 measured by ELISA from lung homogenates under papain treatment. **(I)** Plot shows the expression of *Ifng* lung homogenates of control and Smo-inhibitor groups from PBS and papain protocol by QRT-PCR. **(J)** Expression of *Muc5ac* from lungs under different conditions. **(K)** Representative images showing cellular infiltration in H&E-stained lung sections from control and Smo-inhibitor groups following the papain protocol, ×20 objective lens (Plan-Neofluar/0.5NA). Bar chart: mean ± SEM score on scale of cellular infiltration of airways, from 0 (normal) to 3 (strong infiltration) (arbitrary units) (n = 3). Scale bar: 50 µm. For PBS groups n = 5 and papain-treated groups n = 6; for **(F, I)**: PBS groups n = 5; papain-treated group n = 3; *p < 0.05, **p < 0.01 unpaired Student’s t-test. Data shown are from a representative experiment, and n numbers represent individual mice (biological replicates) in the experiment shown. n.s., not significant.

Mice were exposed to papain protease (Sigma) in phosphate-buffered saline (PBS) or PBS alone (control), applied drop-wise to the nose while under isoflurane-induced anesthesia. This sequence of treatments with papain is referred to as the papain protocol in the manuscript and is shown in **Figure 1A**. Additionally, PF-04449913 (Smo inhibitor) (19), provided by Pfizer, was dissolved in DMSO at a concentration of 3.47 mg/ml. Mice received a daily intraperitoneal injection of 40 µg Smo inhibitor in 200 µl of PBS. Control mice received DMSO in PBS at the same concentration as that of the Smo-inhibitor group. At day 10, mice were sacrificed and bronchioalveolar lavage (BAL), lungs, and mediastinal lymph nodes (mLN) were collected. All experiments were repeated at least twice.

### Cell Isolation

BAL was collected by lavaging the airway four times with 1 ml of PBS + 0.01% EDTA. Lung tissue was mechanically chopped and incubated in digestion cocktail (DMEM medium containing Liberase 250 μg/ml and DNase 1 0.5 mg/ml) at 37°C for 30 min and then subjected to erythrocyte lysis for flow cytometry or subjected to lysis for RNA extraction. The lung was homogenized to obtain whole-lung supernatants for cytokine analysis. A cell suspension was made of mLN harvested for flow cytometry.

### Quantitative RT-PCR

The Absolutely RNA Miniprep Kit (Agilent) was used for extraction of RNA from lung homogenates. Following the manufacturer’s guidelines, cDNA was synthesized using the High-Capacity cDNA Reverse Transcription Kit (Applied Biosystems) and analyzed on an iCycler (Bio-Rad Laboratories, Hercules, CA, USA) using SYBR Green Supermix (Bio-Rad). The housekeeping gene *Hprt* was used for quantification of template and normalization of each gene, as described (36). RT-PCR primers were purchased from Qiagen (QuantiTect primer assay).

### ELISA

IL-13, IL-4, and serum IgE were measured using a Ready-Set-Go! Kit (eBioscience) according to the manufacturer’s instructions.

### Antibodies and Flow Cytometry

Cell suspensions were made and cells stained as described (25, 37) using the following directly conjugated antibodies from BioLegend unless otherwise stated: CD11b APC (clone M1/70), Siglec-F PE (BD Bioscience, clone E50-2440), CD4 FITC, AF700 (clone GK1.5), ST2 APC (clone DIH9), CD117 APC (ACK2), FcεRI PerCP-eFluor 710 (eBioscience, clone MAR1), CD49b PE (clone DX5), IL4rα PE (clone I015F8), and CD25 (clone 3C7). A minimum of 1 × 10^5^ cells were acquired, and doublets were excluded by gating cells on FSC-H/FSCA, as illustrated in **Supplementary Figure 1**. For intracellular cytokine staining, mLN cells were stimulated with Cell Activation Cocktail (with Brefeldin A, BioLegend) at 1 × 10^6^ cells/ml, incubated in complete RPMI (10% FBS and 5% Pen/Strep) at 37°C for 6 h. Then, cells were fixed using Ic Fixation buffer (Invitrogen) and permeabilized using Permeabilization Buffer (Invitrogen) to carry out the intracellular staining for IL-4 PE (clone 11B11), IL-13 Pecy7 (Invitrogen, clone eBio13A), and IFN-*γ* FITC (clone XMG1.2). Cells (2 × 10^6^) were used for transcription factor staining. Staining against Foxp3 PE and Gata3 PE was performed using the Transcription Factor Staining Buffer Set according to the manufacturer’s instructions. Data were acquired on a C6 Accuri flow cytometer (BD Biosciences) or Cytoflex (Beckman) and analyzed using FlowJo v10.6.

### Histological Examination

Lung tissues as formalin-fixed paraffin-embedded samples sectioned for H&E staining were processed and analyzed as described (5). Cellular infiltration was scored blind: 0, normal aspect; 1, mild infiltration around airway; 2, moderate infiltration around airway; 3, strong infiltration around airway.

### Immunofluorescence

Immunofluorescence was performed on fresh frozen acetone-fixed 7-µm sections of OCT-embedded tissue. Sections were blocked for non-specific binding. To detect Shh, goat anti-Shh (1:50; N-19; Santa Cruz Biotechnology) was added overnight, followed by antibody donkey anti-goat IgG Alexa Fluor 594 (1:1,000; Invitrogen). Slides were mounted with Gold Antifade reagent with DAPI (Invitrogen) and visualized using ×40 magnification in Zeiss Observer. Image analysis was performed using Fiji software.

### Statistical Analysis

Unpaired Student’s t test was used for statistical analysis of mouse experiments. Probabilities were considered significant if *p*<0.05(*), *p*<0.01(**).

## Results

### Systemic Smoothened-Inhibitor Treatment Reduced Expression of Hh Pathway Components and the Inflammatory Response and Upon Allergic Sensitization

To investigate the impact of systemic pharmacological Smo inhibition on disease severity in a mouse model of airway inflammation, we used papain, a potent allergen that has been linked to human occupational allergy (18), which is used in mouse models to induce allergic airway inflammation and the Th2 response (10, 38, 39). Papain was administered to mice as described previously, with some modifications: intranasal papain administration was carried out on day 0 (25 µg) day 7 (15 µg), and 9 (15 µg). PBS solution was administrated as control. Mice received intraperitoneal injections of either Smo inhibitor or DMSO (control) daily throughout induction of allergic airway disease following the papain protocol illustrated in **Figure 1A**. Animals were sacrificed and analyzed on day 10.

As Shh expression in lung is increased in allergic asthma (4–6), we first tested if Smo-inhibitor treatment influenced Shh expression on disease induction. Immunofluorescence staining showed that under PBS conditions, there was a low expression of Shh in lung from both Smo-inhibitor-treated and control mice. However, after allergic sensitization by papain treatment Shh expression was increased around lung structures including bronchial/bronchiolar epithelium (**Figure 1B**). After papain treatment, expression of Shh was lower in Smo-inhibitor-treated mice compared to controls (**Figure 1B**), and *Shh* and *Smo* mRNA expressions in lung tissue were also lower in the Smo-inhibitor-treated group (**Figure 1C**), whereas there was no difference in the levels of *Ptch1* transcripts between the groups (**Figure 1C**).

We evaluated the allergic inflammatory process in Smo-inhibitor and control groups of mice. Levels of IgE in serum from PBS-treated Smo-inhibitor and control mice were indistinguishable. The papain-protocol increased serum IgE, indicating induction of an allergic response, and Smo-inhibitor treatment significantly decreased the serum IgE concentration compared to control (**Figure 1D**). We also measured the level of expression of Th2 cell cytokines that coordinate allergic inflammation. Papain treatment increased the expression levels of *Il4* and *Il13* in lung tissue. Smo-inhibitor treatment led to significantly lower levels of transcripts of *Il4* and *Il13* in lung tissue (**Figures 1E, F**). Likewise, protein levels of IL-4 and IL-13 in lung were lower in the Smo-inhibitor group compared to control at the end of the papain protocol (**Figures 1G, H**). IFN-*γ* has been shown to decrease airway inflammation by inhibiting Th2 response in the lung (40), but we did not find significant differences in *Ifng* expression under any treatment (**Figure 1I**). As mucous production is increased in allergic asthma, we examined mucin gene expression (*Muc5ac*) by QRT-PCR from lung homogenates, as a measure of mucus hypersecretion. The expression of *Muc5ac* was markedly lower in Smo-inhibitor-treated mice compared to controls at the end of the papain protocol (**Figure 1J**). After papain treatment, histological analysis of lung tissue showed that Smo-inhibitor treatment significantly lowered cellular infiltration compared to control (**Figure 1K**).

**Figure 2:**
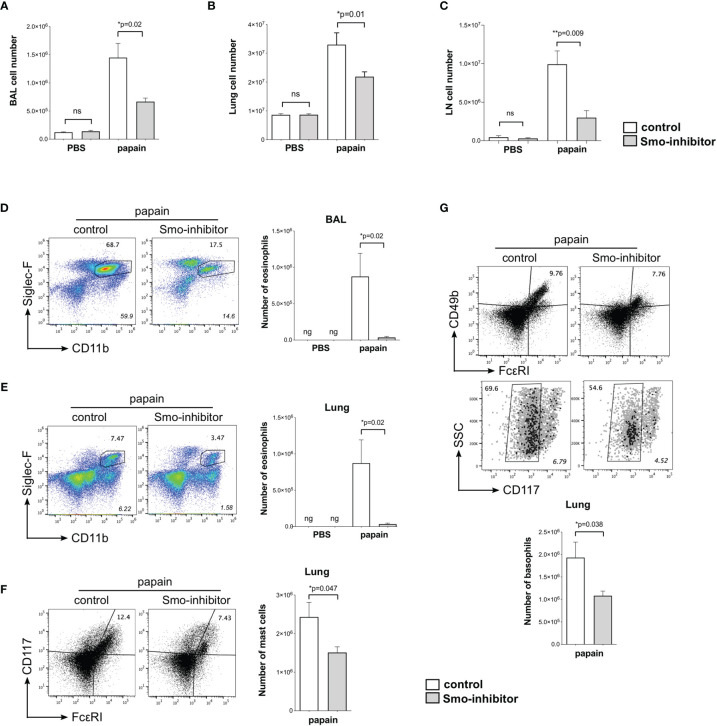
Reduction of inflammatory response upon allergic sensitization by systemic smoothened-inhibitor treatment. Mice underwent the PBS or papain protocol and smoothened inhibitor or control treatment as in **Figure 1A**. **(A–C)** Bar charts: mean ± SEM number of cells recovered from **(A)** BAL, **(B)** lung, and **(C)** mLN under different treatments. **(D,E)** Analysis of eosinophils, giving percentage of eosinophils, from **(D)** BAL and **(E)** lung from control and Smo-inhibitor conditions under papain treatment. Italicized numbers indicate percentage of eosinophils in the live gate. Bar chart: mean ± SEM number of eosinophils under different treatments. **(F)** Flow cytometry: cells from lung stained against CD117 and FcεRI, showing the percentage of CD117^+^FcεRI^+^ (mast cells) from control and Smo-inhibitor papain-treated groups. Bar chart: mean ± SEM number of mast cells. **(G)** Flow cytometry: cells from lung of papain-protocol control and Smo-inhibitor groups to identify basophils (CD49b^+^FcεRI^+^CD117^-^). Non-italicized numbers on plots give the percentage of cells in the regions shown, and italicized numbers indicate percentage of basophils in the live gate. Bar chart: mean ± SEM number of basophils. For **(A, B, D–G)**: PBS groups n = 5 and papain-treated groups n = 6; for **(C)** PBS groups and papain-Smo-inhibitor n = 5; papain-control group n = 6; ng, negligible; *p < 0.05, **p < 0.01, unpaired Student**’**s t-test. Data shown are from a representative experiment, and n numbers represent individual mice in the representative experiment shown. n.s., not significant.

We used flow cytometry to analyze inflammatory and immune cell populations that are important in the development of allergic inflammation. We recovered fewer cells from BAL, lung, and mLN from Smo-inhibitor-treated mice than controls (**Figures 2A–C**). Eosinophil infiltration is a characteristic feature of allergic airway inflammation in mouse models of allergic asthma (41). After the papain protocol, BAL and lungs from Smo-inhibitor-treated mice contained significantly fewer eosinophils (SiglecF^+^CD11b^+^) than control mice (**Figures 2D, E**). Mast cells and basophils are also important inflammatory cells in the development and mediation of the Th2 immune response in allergic asthma (42, 43). Significantly fewer mast cells (FcεRI^+^CD117^+^) and basophils (CD49b(DX5)^+^FcεRI^+^CD117^-^) were isolated from lung in the Smo-inhibitor-treated group than control following the papain protocol (**Figures 2F, G**). Thus, overall Smo-inhibitor treatment reduced the induction of allergic inflammation on papain treatment, lowering serum IgE, expression in the lung of the Th2 cytokines IL-4 and IL-13, and cellular infiltration of eosinophils, basophils, and mast cells to the lung.

### Administration of Smo Inhibitor Decreases CD4 Populations

**Figure 3:**
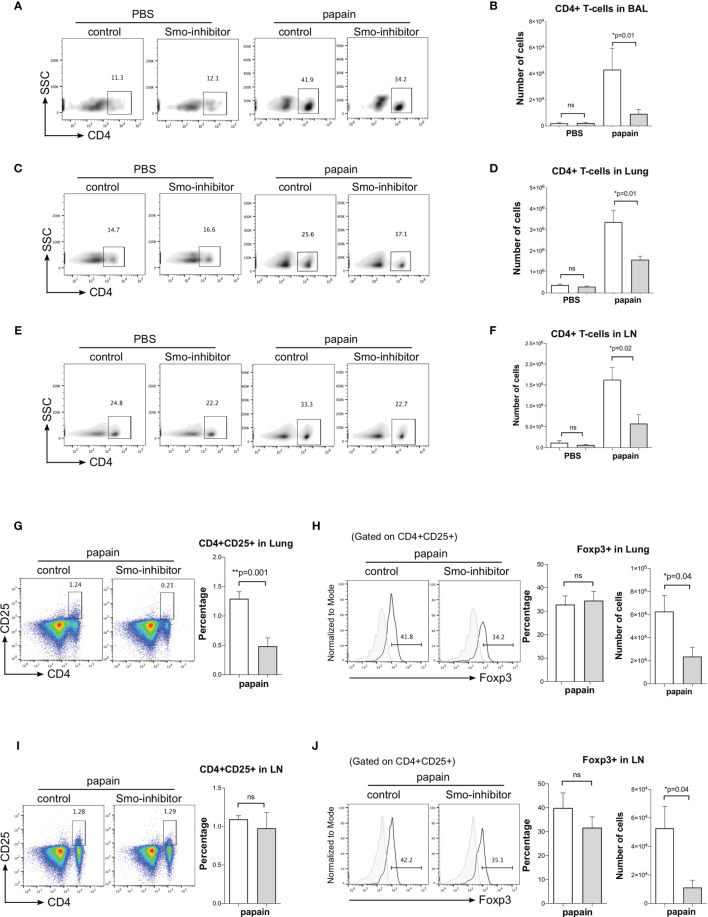
Administration of Smo inhibitor decreases CD4+ T-cells upon papain challenge. Mice underwent the PBS or papain protocol and Smoothened inhibitor or control treatment as in **Figure 1A**. **(A–F)** Facs analysis of CD4+ T-cells under different treatments. Density plots: anti-CD4 staining of cells from **(A)** BAL **(C)** lung, and **(D)** mLN, giving percentage of cells in the region. Bar charts: mean ± SEM number of CD4+ T-cells in **(B)** BAL, **(D)** lung, and **(F)** mLN from PBS and papain-protocol mice treated with Smo inhibitor and control. **(G–J)** Facs analysis of Treg populations in lung and mLN. **(G)** Representative Facs plots show anti-CD4 and anti-CD25 staining in lung under papain conditions. Bar chart: mean ± SEM percentage of CD4+CD25+ T-cells under papain conditions. **(H)** Histograms show intracellular expression of Foxp3 in CD4+CD25+ lung cells in the papain group. Bar charts: mean ± SEM (left) percentage and (right) number of Foxp3+CD4+CD25+ in lung cells under papain treatment. **(I)** Facs plots show anti-CD4 and anti-CD25 staining in mLN under papain conditions. Bar chart: mean ± SEM percentage of CD4+CD25+ under papain conditions. **(J)** Histograms show intracellular expression of Foxp3 in CD4+CD25+ lung cells in the papain group. Bar charts: mean ± SEM (left) percentage and (right) number of Foxp3+CD4+CD25+ in mLN under papain treatment. Gray histograms show staining with an isotype control antibody. PBS groups and papain-Smo inhibitor n = 5; papain-control group n = 6; *p < 0.05, **p < 0.01 unpaired Student**’**s t-test. Data shown are from a representative experiment, and n numbers represent individual mice in the experiment shown. n.s., not significant.

We then analyzed CD4 T-cell populations under different treatments. Under PBS conditions, Smo-inhibitor treatment had no effect on the number of CD4 T-cells in BAL, lung, and mLN. As expected, papain treatment increased the number of CD4 T-cells in BAL, lung, and mLN. However, the number of CD4+ T-cells in BAL, lung, and mLN from the papain-protocol Smo-inhibitor-treated mice was significantly lower than in papain-treated controls (**Figures 3A–F**).

Treg cells (CD4+CD25+Foxp3+) are essential for immune homeostasis and regulation. In allergic airway disease, it has been reporter that Tregs might suppress inflammation and progression of asthma (44). Therefore, to test if the reduction in lung inflammation observed on Smo-inhibitor treatment might be the result of an increase in Tregs, we analyzed the Treg populations in lung and mLN. The percentage of CD4+CD25+ T-cells was lower in the lung of Smo-inhibitor-treated mice compared to control following papain treatment (**Figure 3G**), consistent with the overall reduction in CD4+ T-cells in the lung (**Figure 3C**). However, gating on lung CD4+CD25+ cells, we detected no significant difference in the percentage of cells that expressed Foxp3 between Smo-inhibitor-treated and control groups at the end of the papain protocol (**Figure 3H**). Consistent with the reduction in the overall number of CD4+ T-cells in the lung of papain protocol mice on Smo-inhibitor treatment, the number of lung CD4+CD25+Foxp3+ cells was significantly reduced compared to control (**Figure 3H**). In the mLN, there was no difference in the percentage of CD4+CD25+ cells and in the expression of Foxp3 by the CD4+CD25+ population in the Smo-inhibitor group compared to control under papain conditions, but there was a decrease in the number of the CD4+CD25+Foxp3+ cells, consistent with overall reduction in the number of CD4+ T-cells (**Figures 3I, J**). Thus, we did not observe an increase in Tregs that might account for the decrease in inflammation observed in the papain-treated Smo-inhibitor group. In both lung and mLN, the reduction in the number of Treg was in accordance with the overall reduction in the CD4+ T-cell count, and also consistent with previous reports that Smo inhibition or Shh treatment can reduce or increase the Treg population respectively in other tissues (15, 28).

**Figure 4:**
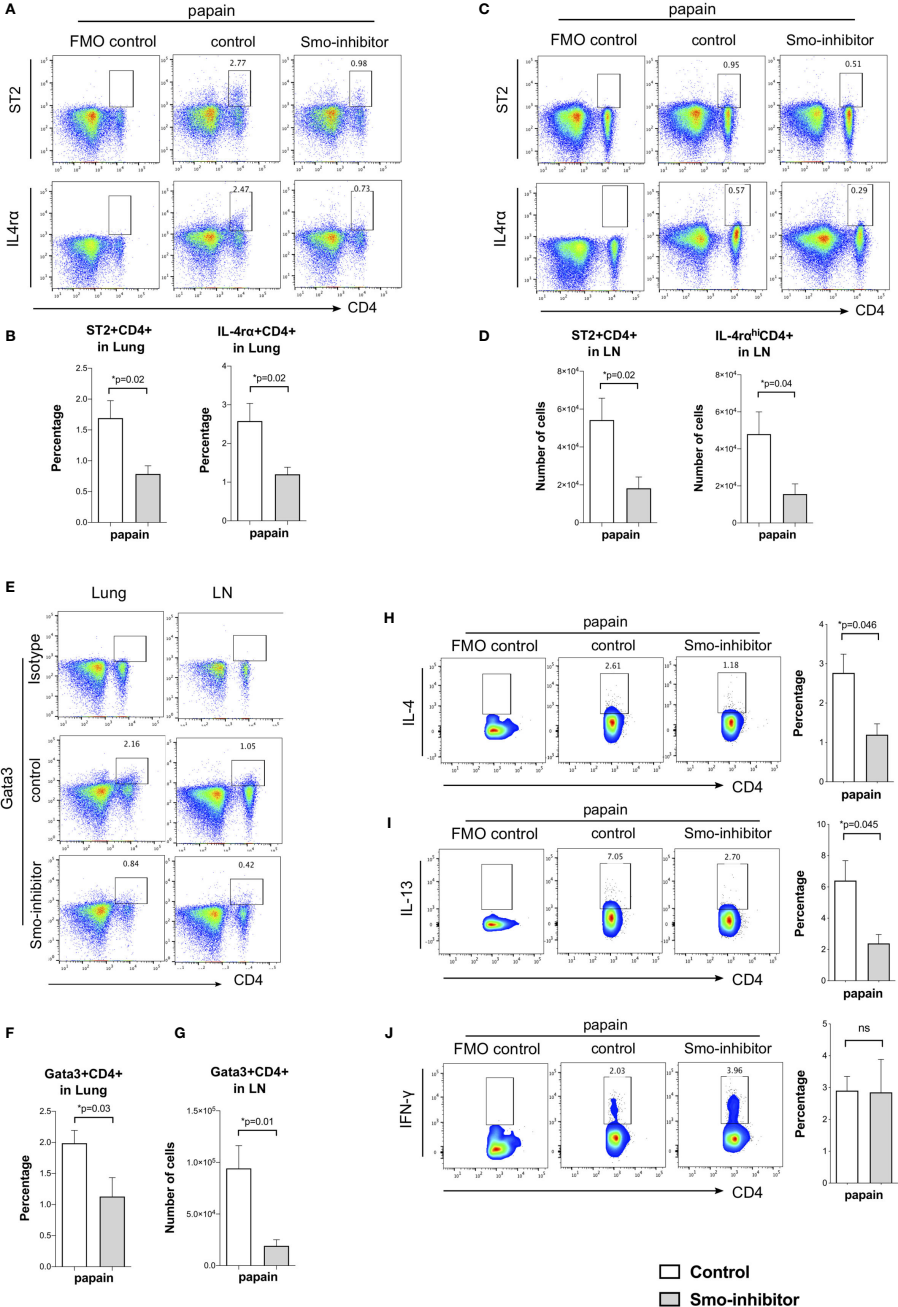
Smo-inhibitor treatment decreased Th2 differentiation and Th2 inflammation in the murine allergic airway model. **(A–D)** Facs analysis of Th2 markers in CD4 T-cells from lung and mLN under papain conditions. Facs plots show anti-CD4 and anti-ST2 (upper plots); anti-CD4 and anti-IL-4rα (lower plots) in **(A)** lung and **(C)** mLN for control and Smo-inhibitor groups from papain-protocol mice. **(B)** Bar charts: mean ± SEM percentage of ST2+CD4+ and IL-4rα+CD4+ T-cells recovered from lung under different treatments. In each case, the control plots show negative control. **(D)** Bar charts: from mLN mean ± SEM number of ST2+CD4+ and IL-4rα^hi^CD4+ T-cells under papain treatment. **(E)** Representative Facs plots of CD4 and intracellular Gata3 staining in lung (left) and mLN (right) under papain treatment. In each case, the control plots show staining with an isotype control antibody. **(F)** Bar chart shows the mean ± SEM percentage of Gata3+CD4+ cells in lung under papain treatment. **(G)** Bar chart shows mean ± SEM number of Gata3+CD4+ cells in mLN under papain treatment (n = 4). **(H)** Facs plots (left) show anti-CD4 and anti-intracellular IL-4 staining in mLN gated on CD4+ T-cells in control and Smo-inhibitor groups under papain group treatment. Bar chart shows mean ± SEM percentage of cells that stained positive for IL-4 in CD4+ T-cells in the control and Smo-inhibitor groups under papain treatment (n = 3). **(I)** Facs plots (left) show anti-CD4 and anti-intracellular IL-13 staining in mLN gated on CD4+ T-cells in control and Smo-inhibitor groups under papain group treatment. Bar chart (right) shows mean ± SEM percentage of CD4 T-cells recovered that stained positive for IL-13 in control and Smo-inhibitor groups under papain treatment (n = 3). **(J)** Facs plots (left) show anti-CD4 and anti-intracellular IFN-*γ* staining in mLN gated on CD4+ T-cells in control and Smo-inhibitor groups under papain group treatment. Bar chart (right) shows the mean ± SEM percentage of CD4 T-cells that stained positive for IFN-*γ* in control and Smo-inhibitor groups after papain treatment (n = 3). In each case, the control plots show negative control. For **(A–D, F)**: PBS groups and papain-Smo inhibitor n = 5; papain-control group n = 6; *p < 0.05 unpaired Student**’**s t-test. Data shown are from a representative experiment, and n numbers represent individual mice in the experiment shown. n.s., not significant.

Analysis of markers of CD4+ Th2 differentiation showed that Smo inhibition significantly reduced Th2 differentiation of lung and mLN CD4+ T-cells in allergic airway disease. At the end of the papain protocol in lung and mLN, the proportion of CD4+ T-cells that expressed ST2 and IL-4rα receptors was significantly reduced in the Smo-inhibitor-treated group compared to control (**Figures 4A–D**). This indicated a reduction in the Th2 effector subset after Smo-inhibitor treatment, as IL-4 is a key Th2 cytokine, and ST2 is an IL-33 receptor that is involved in the Th2 inflammatory response and asthma, which can also be used as a marker of Th2 identity (45, 46). Likewise, we analyzed the expression of the key Th2 transcription factor, Gata3. We found a decrease in Gata3+CD4+ T-cells in the lung and mLN in the Smo-inhibitor group compared to control under papain treatment (**Figures 4E–G**). We finally analyzed the expression of the main Th2 cytokines IL-4 and IL-13 in the mLN CD4 T-cells. Consistent with the lower expression of IL-4 and IL-13 in the lung, and the reduction in CD4 T-cell infiltration and Th2 differentiation, Smo-inhibitor treatment led to a significantly lower expression of IL-4 and IL-13 in the mLN CD4+ T-cells after papain treatment in the Smo-inhibitor treatment group compared to the control (**Figures 4H, I**). There were no significant differences in the percentage of CD4+IFN-*γ* T-cells from mLN under any treatment (**Figure 4J**).

## Discussion

These experiments showed that systemic pharmacological Smo inhibition led to lower T-cell infiltration and a reduction in Th2 cells in the lung and was protective against allergic airway disease, reducing inflammation, and expression of the Mucin gene *Muc5ac* and serum IgE. A recent study examined the effect of intranasal treatment with Hh inhibitors (neutralizing anti-Shh monoclonal antibodies and cyclopamine) in a model in which mice were sensitized and challenged by aerosolization with ovalbumin (OVA) (6). In that study, Hh-inhibitor treatment after each OVA challenge reduced eosinophils and macrophages in BAL, but lymphocyte numbers were unchanged. In contrast, our experiments show that systemic treatment with the Smo inhibitor not only reduced Shh, IL-4, and IL-13 upregulation in the lung and mLN, and inflammatory cell infiltration to lung and BAL, but also reduced CD4+ T-cell populations in the BAL, lung, and mLN and reduced Th2 differentiation and cytokine production within the CD4+ population.

Thus, our study showed a clear and measurable impact of Smo inhibition in allergic airway disease on many aspects of airway inflammation and also a significant reduction in the conventional CD4+ Th2 effector subset. However, further studies will be required to investigate the cellular mechanisms that lead to less severe disease and inflammation and in particular to investigate the impact of Smo inhibition on innate lymphoid cells group 2 (ILC2). ILC2 are tissue-resident cells that are able to secrete Th2 cytokines in response to type 2 alarmins (47). Like Th2 cells of the adaptive immune system, ILC2 have been shown to be important in initiating and maintaining type 2 immune responses in papain-induced lung inflammation models (48–50). The role of Hh signaling in the differentiation and function of ILC2 is currently unknown, so further work is needed to explore this and to investigate whether Hh signaling influences the link between innate and the adaptive responses mediated by ILC2.

Systemic Smo-inhibitor treatment in mice can also influence other T-cell subsets in different tissues. In thymus and spleen, systemic Smo-inhibitor treatment reduced *γ*δ T-cell and *γ*δNKT cell populations (14). In skin, on induction of allergic atopic dermatitis (AD), systemic Smo-inhibitor treatment increased skin inflammation, swelling, and IgE production, but reduced Tregs and Shh expression (15). Thus, the difference in outcomes between Smo inhibition in models of allergic disease in lung and skin would appear to be the result of the different effects of lowering Shh expression in the two tissues: in lung, Shh signals to T-cells to promote Th2 differentiation and function driving allergic asthma, so that reduction in its expression ameliorates allergic disease, whereas in skin Shh signals to induce regulatory T-cell function and so its upregulation is protective against inflammation and disease, and Smo inhibition aggravates it (4, 5, 15). The reason why Shh signaling should affect T-cells differently in lung and skin is unknown and will require further research. It may reflect differences in the Shh signal strength in lung and skin, or be the result of other external signals that T-cells receive in each environment, or of intracellular differences (state of activation or differentiation) between T-cells in the different tissues at the time of Shh signaling.

In conclusion, our study suggests that targeting Shh signaling might be a useful approach to prevent or reduce allergic airway inflammation, but given the tissue-dependent differences in outcome of inhibiting Hh signaling in atopic diseases of skin and lung, and the fact that susceptible individuals may exhibit several different sites of allergic inflammation, more research is needed to understand the way in which Shh secretion in different barrier tissues influences T-cell differentiation and function.

## Data Availability Statement

The original contributions presented in the study are included in the article/**Supplementary Material**. Further inquiries can be directed to the corresponding author.

## Ethics Statement

The animal study was reviewed and approved by the UCL ethics committee.

## Author Contributions

DY and TC conceived the study, designed the experiments, and wrote the manuscript. DY, EP, MC, JR, C-IL, and SR performed and analyzed the experiments. All authors critically reviewed the manuscript. All authors contributed to the article and approved the submitted version.

## Funding

This work was funded by the MRC (MR/P000843/1; MR/5037764/1) and BBSRC (BB/T020970/1). MC was supported by an MRC industrial case studentship and JR by a studentship from the BBSRC London Interdisciplinary Biosciences Consortium (LiDO).

## Conflict of Interest

The authors declare that the research was conducted in the absence of any commercial or financial relationships that could be construed as a potential conflict of interest.

## Publisher’s Note

All claims expressed in this article are solely those of the authors and do not necessarily represent those of their affiliated organizations, or those of the publisher, the editors and the reviewers. Any product that may be evaluated in this article, or claim that may be made by its manufacturer, is not guaranteed or endorsed by the publisher.

## Supplementary Material

Supplementary Figure 1 **(A–E)** Facs plots illustrate the gating strategies. **(A)** BAL and **(B)** lung were gated on live cells using forward (FSC) and side (SSC) scatter. Gated live cells were then analysed using the area FSC-A against height FSC-H to discriminate doublets from singlets. Single cells were subsequently analyzed to identify eosinophils, basophils and mast cells. Gating strategy for T cells. BAL and lung were gated on live cells using forward (FSC) and side (SSC) scatter. Gated live cells were then analysed using the area FSC-A against height FSC-H to discriminate doubles from singlets and then lymphocyte population was identified based in forward (FSC) and side (SSC) scatter to further analysis of CD4 T-cells. **(E)** mLN cells were gated on live cells using forward (FSC) and side (SSC) scatter and gated live cells were analysed using the area FSC-A against height FSC-H to discriminate doublets from singlets and to further analyse CD4 T-cells.
