# Supplementary figures and images for: Systemic Pharmacological Smoothened Inhibition Reduces Lung T-Cell Infiltration and Ameliorates Th2 Inflammation in a Mouse Model of Allergic Airway Disease
Source: Front Immunol. 2021 Sep 10;12:737245. doi: 10.3389/fimmu.2021.737245 (PMC8463265; doi:10.3389/fimmu.2021.737245)

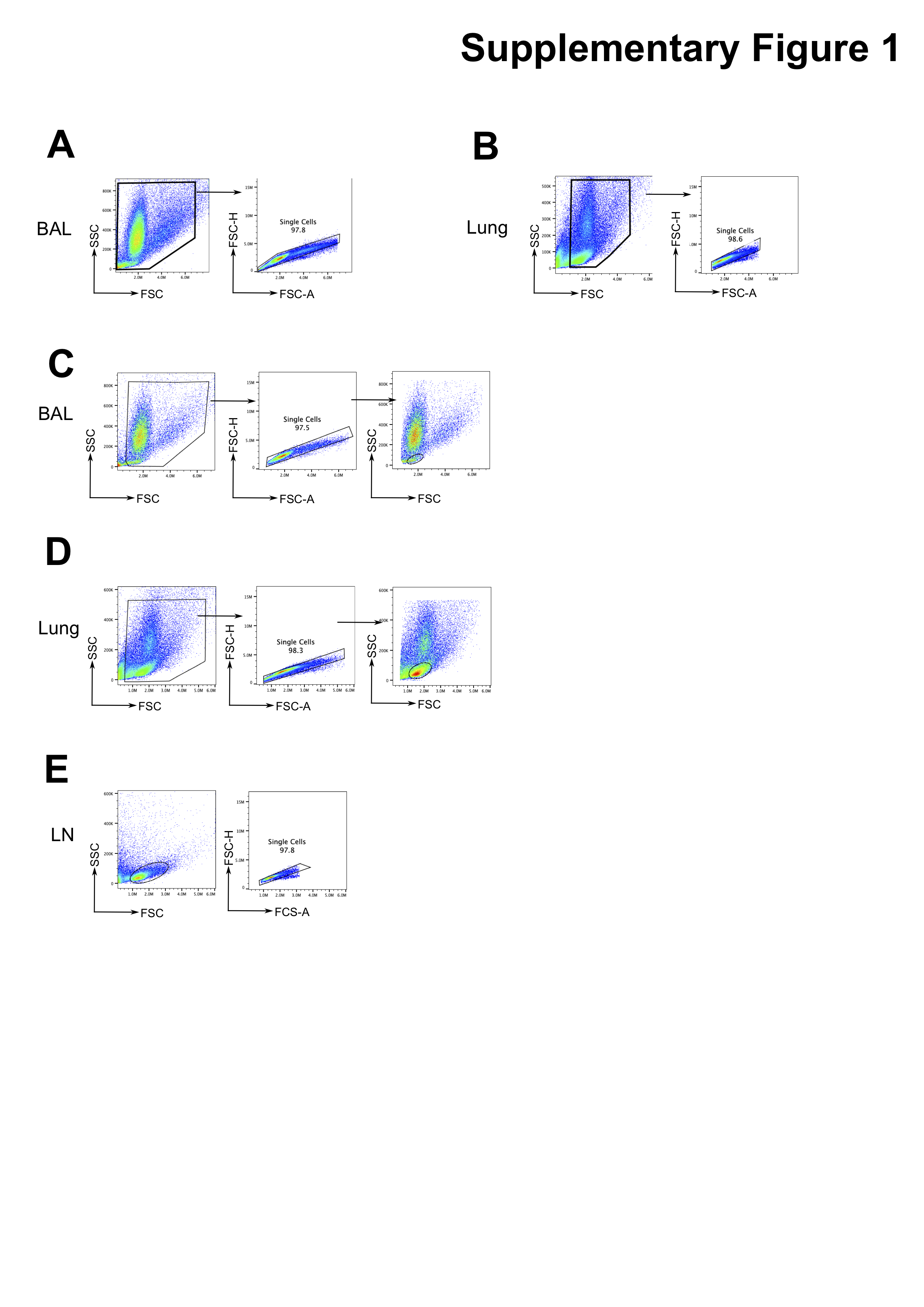

Supplement: Supplementary Figure 1 — (A–E) Facs plots illustrate the gating strategies. (A) BAL and (B) lung were gated on live cells using forward (FSC) and side (SSC) scatter. Gated live cells were then analysed using the area FSC-A against height FSC-H to discriminate doublets from singlets. Single cells were subsequently analyzed to identify eosinophils, basophils and mast cells. Gating strategy for T cells. BAL and lung were gated on live cells using forward (FSC) and side (SSC) scatter. Gated live cells were then analysed using the area FSC-A against height FSC-H to discriminate doubles from singlets and then lymphocyte population was identified based in forward (FSC) and side (SSC) scatter to further analysis of CD4 T-cells. (E) mLN cells were gated on live cells using forward (FSC) and side (SSC) scatter and gated live cells were analysed using the area FSC-A against height FSC-H to discriminate doublets from singlets and to further analyse CD4 T-cells. [file Image_1.jpeg]
