# Supplementary material for: Systemic Pharmacological Smoothened Inhibition Reduces Lung T-Cell Infiltration and Ameliorates Th2 Inflammation in a Mouse Model of Allergic Airway Disease
Source: Front Immunol. 2021 Sep 10;12:737245. doi: 10.3389/fimmu.2021.737245 (PMC8463265; doi:10.3389/fimmu.2021.737245)
Supplement: Supplementary file 2 [file Table_1.docx]

**Supplementary Table 1**: List of reagents and antibodies used in this study
